# Molecular detection and characterisation of Domestic Cat Hepadnavirus (DCH) from blood and liver tissues of cats in Malaysia

**DOI:** 10.1186/s12917-020-02700-0

**Published:** 2021-01-06

**Authors:** Khanmani Anpuanandam, Gayathri Thevi Selvarajah, Mandy Mun Kei Choy, Shing Wei Ng, Kiven Kumar, Razana Mohd Ali, Sujey Kumar Rajendran, Kok Lian Ho, Wen Siang Tan

**Affiliations:** 1 Department of Veterinary Clinical Studies, Faculty of Veterinary Medicine, Universiti Putra Malaysia, Selangor 43400 UPM Serdang, Malaysia; 2 Institute of Bioscience, Universiti Putra Malaysia, 43400 UPM Serdang, Selangor Malaysia; 3 Department of Pathology, Faculty of Medicine and Health Sciences, Universiti Putra Malaysia, 43400 UPM Serdang, Selangor Malaysia; 4 Department of Microbiology, Faculty of Biotechnology and Biomolecular Sciences, Universiti Putra Malaysia, 43400 UPM Serdang, Selangor Malaysia

**Keywords:** Hepadnavirus, Liver, PCR, Prevalence, Risk factors, Clinical pathology, Phylogenetic analysis, Feline, Malaysia

## Abstract

**Background:**

A new domestic cat hepadnavirus (DCH, family *Hepadnaviridae*) was first reported from whole blood samples of domestic cats in Australia in 2018, and from cat serum samples in Italy in 2019. The pathogenesis of DCH is unknown, but it was reported in cats with viraemia (6.5–10.8%), chronic hepatitis (43%) and hepatocellular carcinoma (28%). Recent reports suggest that DCH resembles the human hepatitis B virus (HBV) and its related hepatopathies. This study aims to detect and characterize DCH among domestic cats in Malaysia. A cross-sectional study was performed on 253 cats, of which 87 had paired blood and liver samples, entailing whole-genome sequencing and phylogenetic analysis of DCH from a liver tissue sample.

**Results:**

Among the 253 cats included in this study, 12.3% of the whole blood samples tested positive for DCH. The detection rate was significantly higher in pet cats (16.6%, *n* = 24/145) compared to shelter cats (6.5%, *n* = 7/108). Liver tissues showed higher a DCH detection rate (14.9%, *n* = 13/87) compared to blood; 5 out of these 13 cats tested positive for DCH in their paired liver and blood samples. Serum alanine transaminase (ALT) was elevated (> 95 units/L) in 12 out of the 23 DCH-positive cats (52.2%, *p* = 0.012). Whole-genome sequence analysis revealed that the Malaysian DCH strain, with a genome size of 3184 bp, had 98.3% and 97.5% nucleotide identities to the Australian and Italian strains, respectively. The phylogenetic analysis demonstrated that the Malaysian DCH genome was clustered closely to the Australian strain, suggesting that they belong to the same geographically-determined genetic pool (Australasia).

**Conclusions:**

This study provided insights into a Malaysian DCH strain that was detected from a liver tissue. Interestingly, pet cats or cats with elevated ALT were significantly more likely to be DCH positive. Cats with positive DCH detection from liver tissues may not necessarily have viraemia. The impact of this virus on inducing liver diseases in felines warrants further investigation.

## Background

Domestic cat hepadnavirus (DCH) is a novel member of the *Hepadnaviridae*, detected in domestic cats (*Felis catus*) [1–3]. Hepadnaviruses are spherical with a diameter of 42–50 nm. The viral genome is enclosed in an icosahedral capsid, which is enveloped by a lipid bilayer membrane. The viral genome comprises a circular, partially double-stranded DNA molecule [4, 5]. Hepadnaviruses can be classified into five genera according to host: *Orthohepadnavirus* infects mammals, *Avihepadnavirus* infects bird species, *Herpetohepadnavirus* infects amphibians and reptiles, *Metahepadnavirus* and *Parahepadnavirus* infect fish [6–8]. The newly identified DCH has been classified under *Orthohepadnavirus* [1].

The orthohepadnavirus has a DNA genome of approximately 3.2 kb. The genome has four overlapping open reading frames (ORFs), which encode for the surface (S), X, core (C) and polymerase (P) proteins [9]. Several orthohepadnaviruses have been isolated from various primates including humans, gorillas, gibbons, orangutans, woolly monkeys and chimpanzees [10–13]. Orthohepadnaviruses also infect other mammals including woodchucks, ground squirrels, arctic squirrels and bats [14–17].

The well-known prototype species of this family, human HBV, was first discovered in 1966. Approximately 257 million people worldwide are HBV carriers, and HBV causes 887,000 deaths annually, mainly through hepatocellular carcinoma (HCC, 62%) and cirrhosis (29%) [18]. HBV distribution predominates in various geographical regions worldwide with high prevalence reported in Asia, Middle East and Africa. Different HBV genotypes co-circulate in different regions, which increases the rate of HBV genomic recombination [19].

HBV has also been reported as a common co-pathogen in human immunodeficiency virus (HIV)-infected individuals. Chronic HBV infection occurs in up to 10% of HIV patients co-infected with HBV. The immune system of HIV patients has a decreased likelihood of HBV clearance, which leads to higher HBV viral load and the development of chronic HBV infection [20]. As HBV and HIV co-infection is commonly reported in humans, the initial study on DCH detection focused on cats infected with feline immunodeficiency virus (FIV). DCH prevalence among FIV-infected cats was 10%, which was significantly higher than that in cats not infected by FIV (3.2%) [1].

In Italy, cat serum samples sent to diagnostic laboratories under clinical suspicion of infectious diseases revealed a high DCH prevalence of up to 17.8%; of these, 33.3% of cats infected with retroviruses (FIV and feline leukemia virus, FeLV) also had DCH co-infection [2]. Lanave et al. [2] also found that 7 of 10 suspected hepatic disease cases were also DCH-positive. A feline hepatic lesion-focused study by Pesavento et al. [3], which examined formalin-fixed, paraffin-embedded (FFPE) liver tissues from four countries (the United States, Australia, New Zealand, the UK) demonstrated that up to 43% of chronic hepatitis cases and 28% of HCC cases were also DCH-positive.

Cats aged 4 to 7 months had higher odds of being DCH-positive, albeit at a *p* = 0.08061, and with no sex predisposition. An *in-situ* hybridization (ISH) study suggested that DCH viral distribution in HCC and chronic hepatitis cases resembled that of HBV-related liver diseases in humans [3]. These findings suggest a possible similar pathogenesis for DCH as HBV, whereby the virus could be involved in chronic liver inflammation leading eventually to carcinogenesis. This hypothesis requires further investigations.

Studies on the detection of DCH in paired liver and blood samples from the same cat have not yet been reported. Paired detection results will increase our understanding of DCH infection at different clinical phases as observed in HBV patients, viz. virus carriers and immune-tolerant patients, and between active and chronic phases. Usually, a higher HBV DNA load in blood is found in acute infection or in the active chronic phase of infection [21, 22].

The discovery of DCH in two different geographical locations revealed two disparate DCH strains: DCH Australia (3187 bp, AUS/2016/Sydney) and DCH Italy (3184 bp, ITA/2018/165 − 83) [1, 2]. The Italian and Australian strains share 97% nucleotide identity [2]. Therefore, determining the complete genome sequence of the Malaysian DCH would provide insights into the genetic variability of this virus.

Here, we report on the molecular prevalence of DCH using conventional PCR assay on both whole-blood and liver tissue samples from shelter and pet domestic cats in Malaysia. Possible risk factors for DCH infection were evaluated, including age, sex, type of ownership, elevation in serum alanine transaminase (ALT) levels, and co-infection with FIV and FeLV. In addition, the complete genome sequence of the local (Malaysian) strain was determined and compared with two DCH strains from Australia and Italy. Phylogenetic analysis was performed to relate the evolution of the Malaysian strain of DCH with those of other hepadnaviruses.

## Results

### Study group

A total of 340 samples, comprising 253 blood and 87 liver samples, were collected. All the liver samples were collected with paired blood samples from shelter cats. From pet cats, only blood samples were collected. A total of 108 (42.7%) cats were from the shelter and the remaining 145 (57.3%) cats were pet cats. Out of the 253 total cats, 132 (52.2%) were male while the remaining 121 (47.8%) were female. The mean and median ages of the cats were 4 and 2 years old with a minimum of one-month and a maximum of 19 years. Of the 253 cats, 90.9% were Domestic Short Hair (*n* = 230). The other breeds consisted of Persian (*n* = 10), British Short Hair (*n* = 3), Domestic Long Hair (*n* = 3), Siamese (*n* = 2), Maine Coon (*n* = 2), American Curl (*n* = 2), and one Norwegian Forest.

The cats were diagnosed with various health conditions, predominantly respiratory diseases (*n* = 35), followed by wounds and skin diseases (*n* = 27), gastrointestinal diseases (*n* = 26), traumatic injuries (*n* = 14), neoplasia (*n* = 4), and urogenital diseases (*n* = 42). Cats with poor body condition (*n* = 73) and with systemic illnesses (anaemia, dehydration, general weakness, and anorexia) were also included in this study. Samples were also collected from healthy cats (*n* = 32) undergoing elective procedures such as neutering and general wellness check-ups. The four neoplasia cases included one each of fibrosarcoma, squamous cell carcinoma, lymphoma and mammary adenocarcinoma.

### Molecular detection

**Fig. 1:**
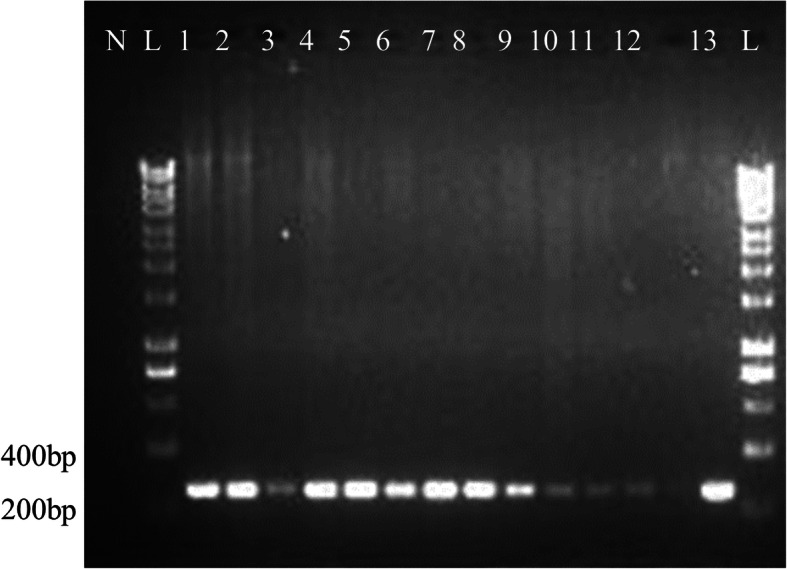
Agarose gel electrophoresis of liver samples. PCR of the *Hgap* gene produced a product of 230 bp. Electrophoresis was performed on 1.5% (w/v) agarose gel. Lane L: 1kb DNA ladder, Lane N: negative control. Lanes 1 to 13: positive samples. Liver sample ID: FH4, FH5, FH13, FH14, FH32, FH41, FH42, FH43, FH45, FH59, FH62, FH246 and FH248

Among the 253 cat blood samples, 12.3% (*n* = 31/253) tested positive for DCH. Among the 87 cat liver tissue samples, 14.9% (*n* = 13/87) tested DCH-positive. Of the 13 cats with DCH-positive liver tissues (Fig. 1), only five (38.5%) were viraemic.

**Fig. 2:**
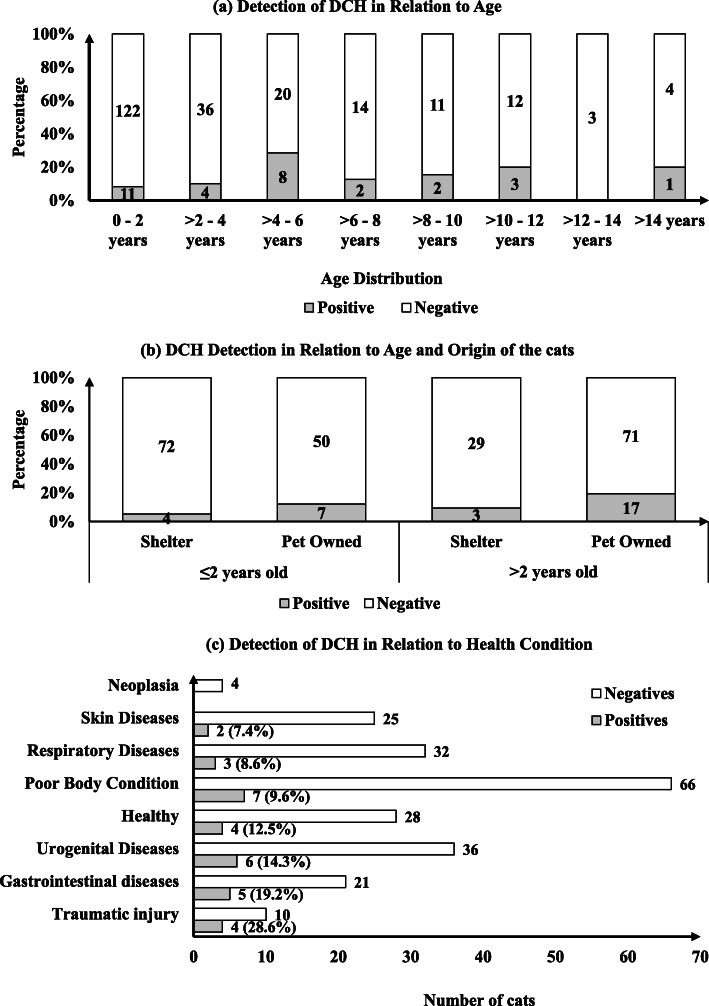
**a** Age distribution of cats in relation to detection of DCH included in this study. Highest percentage of DCH detection was seen in age group of 4 to 6 years old. **b** DCH detection in relation to origin and age group. **c** Health conditions of cats in relation to detection of DCH. Among the traumatic injury cases, the highest percentage of detection was seen

We analysed the distribution of DCH across different age groups (Fig. 2a). Cats older than 2 years had significantly higher odds of having DCH-positive blood samples than the younger cats (*p* = 0.042, CI: 1.015–4.848) (Fig. 2b). Twenty of 31 cats with DCH-positive blood samples were over 2 years of age (64.5%, *n* = 20/31). DCH was detected in more pet cats (16.6%) than shelter cats (6.5%, Table 1). Breed and sex were not risk factors for DCH. Among the different health conditions, the highest detection (28.6%) of DCH was seen in traumatic injury cases (Fig. 2c). The detection of DCH was 12.2% (27/221) in sick cats and 12.5% (4/32) in healthy cats.

### Serum biochemistry

Twelve of the 23 DCH-positive blood samples (52.2%) had elevated ALT levels. Cats with abnormal ALT had a 21% chance of having a DCH-positive blood sample (*p* = 0.012, CI: 1.239–7.294) (Table 1). When DCH was detected in the blood, the odds of having elevated ALT was 6.3 times higher than when DCH was detected solely in the liver tissue. Other serum biochemistry readings, such as alkaline phosphatase (ALP), urea and creatinine, were not significantly associated with detecting DCH.

### Co-infection with feline immunodeficiency virus and feline leukemia virus

DCH was detected as a co-pathogen of the two common feline viruses examined in this study. Four out of five FeLV-positive cats were DCH-positive, which was significant at *p* = 0.006 (CI: 1.352–121.212) (Table 1), but the number of FeLV-positive cats (*n* = 5) was too low to estimate the risk of FeLV patients being coinfected with DCH. One of the two FIV-positive cats was DCH-positive with no significant association.

**Table 1:** *Chi*-square analyses and univariate logistic regression for the risk of DCH in Malaysian cats

Factors	Categories	Prevalence	*P* value	OR	95% CI
Paired Sample	Positive	5/13 (38.5%)	**< 0.001**	0.098	0.051–0.188
	Negative	0/74 (0%)*			
Origin	Shelter	7/108 (6.48%)*	**0.016**	2.862	1.184–6.916
	Pet Cats	24/145 (16.55%)			
Age Class	< 2 years old	11/133 (8.27%)*	**0.042**	2.218	1.105–4.848
	Adult	20/120 (16.7%)			
Sex	Male	20/132 (15.2%)	0.142	0.56	0.256–1.223
	Female	11/121 (9.1%)*			
ALT	Normal	11/135 (8.15%)*	**0.012**	3.006	1.239–7.294
	Abnormal	12/57 (21.05%)			
ALP	Normal	22/169 (13.02%)	0.966	0.955	0.112–8.135
	Abnormal	1/8 (12.5%)*			
Urea	Normal	17/132 (12.88%)	0.725	0.846	0.332–2.157
	Abnormal	7/63 (11.11%)*			
Creatinine	Normal	20/168 (11.9%)*	0.563	1.41	0.439–4.527
	Abnormal	4/25 (16%)			
FIV	Positive	1/2 (50%)	0.458	2.783	0.167–46.333
	Negative	23/87 (26.44%)*			
FeLV	Positive	4/5 (80%)	**0.006**	12.8	1.352–121.212
	Negative	20/84 (23.81%)*			

*OR *Odds ratio, *OR* Odds ratio, *CI* Confidence interval, *Reference for analysis, numbers in bold indicate *p*<0.05, *ALT* Alanine transferase, *ALP* Alkaline phosphatase, *FIV* Feline immunodeficiency virus, FeLV Feline leukemia virus

### Complete genome sequence and phylogenetic analysis of Malaysian DCH

**Fig. 3:**
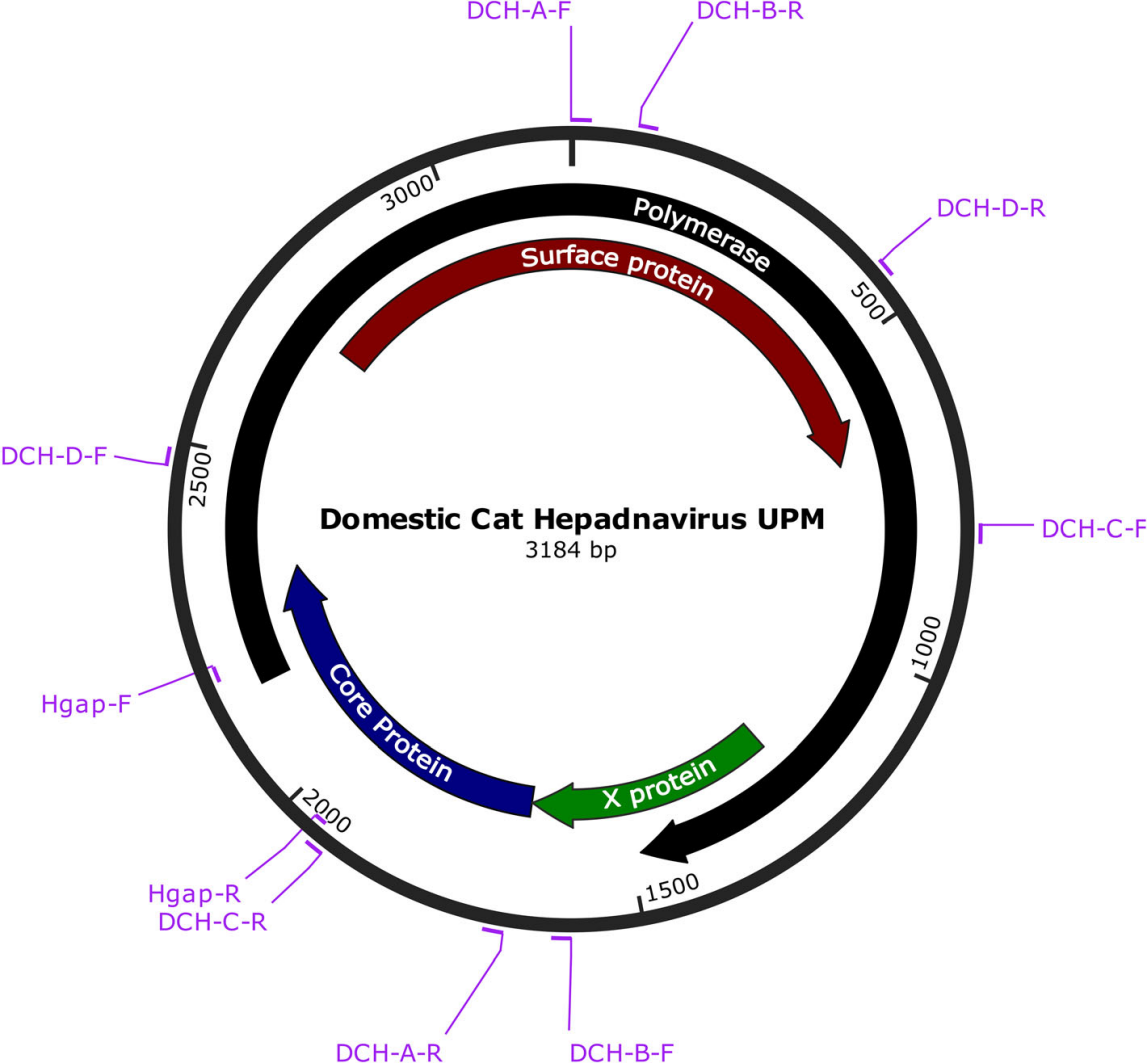
Genomic structure of DCH Malaysia, with the positions of the primers used in this study. The complete genome sequence consists of 3184 bp. The colours indicate the ORFs of polymerase (black), surface protein (red), core protein (blue) and X protein (green)

The complete genome sequence of a local DCH strain was 3184 bp; Fig. 3 illustrates its key genomic features. Complete sequencing was performed for the liver tissue of a cat which was positive for the *Hgap* gene. Similar to Australian and Italian isolates, the genome of the local DCH strain also had four ORFs coding for the P, S, C and X proteins. However, the *P* and *S* genes of the local isolate were each shorter than those of the Australian strain by one codon. Identical to that of the Italian DCH, amino acid deletions were also observed at positions 271 (Pro) and 82 (Ser) in the P and S proteins, respectively, in Malaysian strain. The complete genome sequence has been deposited in GenBank (accession no. MK902920).

**Fig. 4:**
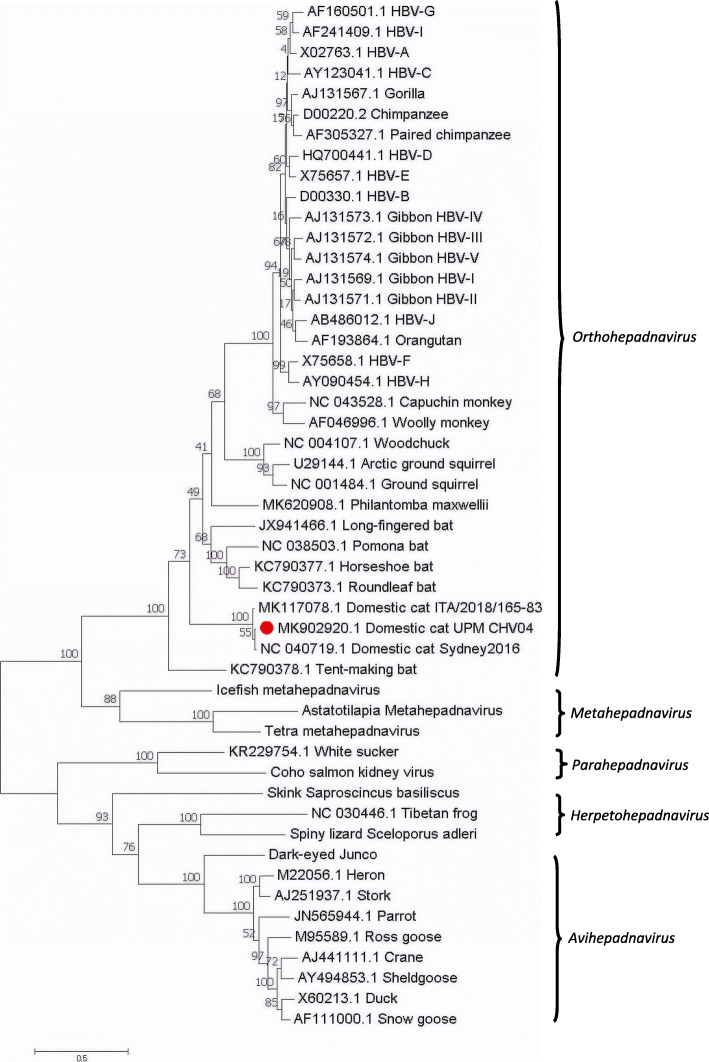
The phylogenetic position of DCH Malaysia within the family *Hepadnaviridae*. Maximum likelihood phylogeny based on the complete genome sequences of a wide range of vertebrate hepadnaviruses retrieved from the Genbank database. The herpetohepadnaviruses, parahepadnaviruses and metahepadnaviruses were described in Dill et al. [8] and Lauber et al. [7]. The DCH Malaysia (Domestic cat hepadnavirus UPM MK902920) is indicated by a red dot. The tree was drawn to scale, with branch lengths measured in the number of substitutions per site

The Malaysian DCH strain was 98.34% genomic nucleotide sequence-identical to the Australian reference strain, with 98.09%, 100%, 98.69% and 96.55% sequence identities for the P, C, S and X proteins, respectively. Figure 4 shows the phylogenetic tree constructed using the maximum likelihood method from the complete genome sequences of DCH and other orthohepadnaviruses, avihepadnaviruses, herpetohepadnaviruses, metahepadnaviruses and parahepadnaviruses. The result revealed that the abovementioned viruses were largely clustered according to the genera. It is worth noting that the Malaysian DCH formed a sister branch with Australian DCH and diverged from Italian DCH.

## Discussion

The present study demonstrated a higher prevalence of DCH in liver tissue samples than in whole-blood samples of domestic cats in Malaysia. Positive detection of DCH had previously been reported in the whole blood, serum, and FFPE liver tissues of cats diagnosed with hepatitis or HCC [1–3]. Interestingly, the findings of this study indicated that DCH detection using liver samples might show the true prevalence of DCH among domestic cats rather than by using blood samples.

The prevalence of DCH in the whole-blood samples in the present study was greater than reported in Italy and Australia [1, 2]. The observed differences could be due to the variations between regions and sample densities. The highest percentage of DCH detection found in traumatic cases could be due to other underlying factors contributing to the injuries in these cats.

Significantly, all the paired blood samples that were DCH-positive also had DCH-positive liver samples; this finding has not been reported before. The hepatocytes of these cats were infected with DCH, but they had no viremia. This could be due to the different stages of infection where the DCH DNA is only detectable in liver tissue.

The prevalence of DCH among Malaysian shelter cats (6.5%) was similar to that among pet cats in Australia (6.5%). However, DCH detection among Malaysian pet cats (16.6%) was notably higher than in Australia and Italy (10.8%), for which the origins of the cats were not mentioned [1, 2]. These observed differences among countries could be due to the sampled populations, geographical variances and control measures.

There is very limited knowledge on the transmission and pathogenesis of DCH. The lack of awareness in the possible transmission routes of this new virus could have led to anecdotally higher detection levels of DCH among pet cats compared to shelter cats. The prototype HBV is transmitted through body fluids and secretions [21, 23]. Whether DCH can be transmitted through the saliva, faeces and urine is still unknown. However, Lanave et al. [2] argued that, since DCH can be detected in the serum and whole blood samples, it is possible for transmission to take place through blood transfusion from one cat to another.

DCH infection did not appear to be significantly linked with sex. This was in good agreement with that reported by Lanave et al. [2] who also detected high DCH prevalence in cats aged 4 to 7 months (20.5%). In contrast, the present study showed a higher DCH prevalence in cats aged > 2 years compared to younger cats. While human HBV can cause different phases of infection prenatally and in young adults and adults [24], the DCH infection phases in cats are still unknown.

The significant association of DCH co-infection with FeLV agrees with Lanave et al. [2], who reported co-infection with both FIV and FeLV. However, separate values for each retrovirus co-infection with DCH were not mentioned in that study. Although the FeLV antigen-positive sample was small in this study, it is important to note that FeLV detection efficay differs between the phases of FeLV infection. The cats are viraemic in the progressive infection phase, when the FeLV antigen is detectable. In the regressive phase, the virus hides in the bone marrow or other tissues, rendering the FeLV antigen undetectable. The FeLV antigen can be reactivated in this phase, and triggers development of clinical signs [25]. The risk of underestimating FeLV prevalence via antigen detection with ELISA hence warrants closer attention; repeated samplings on FeLV-negative cats are normally recommended to verify an initial ELISA result. The low detection of FIV-positive samples in the current study was in line with other FIV-related studies on domestic cats in Malaysia [26, 27].

We found a significant association between elevated ALT and positive DCH detection in blood samples. Twelve of the 57 cats with elevated ALT was DCH-positive, in good agreement with Lanave et al. [2]. For chronic HBV infection, at the immune-tolerant phase, ALT was within the normal range, but high levels of HBV DNA could be detected in the blood, with minimal or no liver inflammation [24]. In the present study, 4 of 11 cats had no elevated ALT although the PCR analysis showed that they were DCH-positive. These findings lead to the possibility of classifying/staging DCH infection in cat patients as in HBV infection. In human patients, staging HBV infection is important for treatment and prognosis purposes. Future investigations using experimental infections of DCH among cats can resolve many unanswered questions on the disease and provide new insights into feline medicine.

This is the first study in Malaysia to explore the presence of DCH among local domestic cats. The total genome sequence of the local strain is similar to that of the Italian strain, missing a single codon from the *P* and *S* genes. However, phylogenetically, the complete genome sequence of the Malaysian strain clustered together with the Australian strain. This could be due to geographical proximity, since Australia and Malaysia are close together, while Italy is much further away. The Malaysian strain is located between the Italian and Australian strains, and more closely related to the Australian strain.

All the three DCH strains are grouped together in a well-defined branch, which is related to several bat, rodent and primate hepadnaviruses. The Malaysian DCH strain is closely related to the previously published Australian DCH strain, suggesting that different strains of DCH may be discovered across different continents. As DCH was not discovered until 2018, many of its features such as pathogenesis, cross-species transmission, and different stages of infections, have yet to be characterized.

## Conclusions

This study provided insights into a Malaysian DCH strain that was detected from a liver tissue. Interestingly, the prevalence of DCH in liver tissue samples was higher than that in blood samples from domestic cats in Malaysia, of which adult and pet cats have a higher prevalence. Nearly 50% of the cats tested positive for DCH showed elevated ALT level. The Malaysian DCH is genetically clustered together with Australian and Italian DCH strains. Overall, our study provided valuable knowledge on the occurrence of DCH in Malaysia, of which the true prevalence can be studied using the liver tissue samples. However, the effect of this virus on causing liver disease in felines warrants further study. As the domestic pet cat population in Malaysia is growing, veterinarians and pet owners need to be advised sufficiently about new infectious diseases and possible zoonosis. Investigations on DCH may also offer new or more effective ways to study HBV.

## Methods

### Sample collection and storage

For this study, whole blood samples were collected from domestic cats from an animal shelter in Selangor and from the University Veterinary Hospital (UVH) of Universiti Putra Malaysia between 2018 and 2019. Blood samples were collected from UVH cat patients with owners’ consent. Blood was sampled from pet cats presented to the UVH for annual health examination, neutering or health issues. Most often blood was drawn as part of the routine diagnostics where left over blood samples were stored for this research. Pet cats were returned to their respective owners. Blood was sampled from healthy cats at the shelter prior to neutering where they were returned to their enclosures.

Cats were physically restrained and blood (about 1 to 2 mL, depending on the cat’s body weight) was drawn via the jugular vein. All blood samples were transferred into a 3 mL plain and EDTA vacutainer tubes (BD Vacutainer®, United States). All blood samples in the plain tube were left standing for one to three hours at room temperature before centrifugation at 2000 *g* for 10 minutes (KUBOTA 2010, Japan). The resultant serum samples were pipetted into 1.5 mL Eppendorf tubes® (USA) and stored at − 20℃. Whole blood samples in EDTA vacutainer tubes were subjected to DNA isolation.

Samples were also collected from cats at the shelter which were euthanized by the shelter veterinarian (not solely for this research purpose) for medical conditions which was beyond the resources of the shelter to treat or due to illnesses of a very contagious or zoonosis in nature. Euthanasia was performed using sodium pentobarbital which was administered intravenously at a dose of > 100 mg/kg. Blood was drawn prior to euthanasia while liver tissues were collected at post-mortem, snap-frozen in liquid nitrogen, and stored in cryotubes at -80 C (SANYO Ultra Low, Japan). Parts of the liver tissue were also placed in 10% (v/v) buffered formalin solution (Sigma-Aldrich, Germany) for routine histopathology. Carcasses were disposed based on the shelter's policy.

### DNA isolation and PCR assay

Total DNA was extracted from 100 µL whole blood using the DNeasy Blood & Tissue Kit (Qiagen, Germany), following the manufacturer’s protocol. DNA was extracted from 25 mg liver tissue, after the tissue was homogenized using a rotor-stator homogenizer and probe (Qiagen). The total DNA from liver tissue was extracted using the DNeasy Blood & Tissue Kit (Qiagen) according to the manufacturer’s protocol.

PCR primers used for detecting the *Hgap* gene to confirm the presence of DCH were synthesized (forward: 5’-GTGCTCTGATAACCGTATGCTC-3’; reverse: 5’-CTAGAATGGCTACATGGGGTTAG-3’) as reported previously [1]. Conventional PCR was performed using the Bioline MyTaq hot-start polymerase (Bioline, Australia) according to the manufacturer’s protocol: 300 ng DNA template, 5 × MyTaq buffer, 20 µM final primer concentration in a total 25 µL reaction. The PCR cycling conditions were as follows: Initial denaturation at 95℃ for 1 min, followed by 40 cycles of denaturation at 95℃ for 15 s, annealing at 55℃ for 15 s, extension at 72℃ for 10 s and final extension at 72℃ for 5 min. The PCR products from both the blood and liver samples were analysed with (1.5%) agarose gel electrophoresis, from which specific bands containing the PCR products were purified, excised and verified. The amplicons were verified using the Sanger DNA sequencing method.

### Complete genome sequencing and phylogenetic analysis of Malaysian DCH

The complete DCH viral genome sequence was obtained from the DCH-positive samples using the four newly designed primers (Table 2). The primers were designed using the National Center for Biotechnology Information (NCBI) Primer-BLAST [28]. Reference was made to the whole-genome sequence of the Australian strain (GenBank, accession no. NC040719). PCR was performed as described above, except that the annealing temperature was as stated in Table 2. The amplicons were analysed using the Sanger sequencing method (Apical, Malaysia). The viral nucleotide sequences were manually edited by removing the overlapping regions to obtain the complete circular genome of the Malaysian DCH. Sequence editing, trimming, reverse complement sequencing and assembly of the forward and reverse sequences were performed using SnapGene® version 4.3 software [29].

**Table 2:** Newly designed primers to amplify the whole genome sequence of DCH

Primer	Sequence	Base pair	Annealing Temperature
**DCH A**	F: 5’-TGGGCAACATTACCTCAGGTCC-3’	1700	56℃
	R: 5’-GGAACAAAAGAGAACGCACAGG-3’		
**DCH B**	F: 5’-GGTCTGACGCCCAGGTTATG-3’	1700	56℃
	R: 5’-ACCACGAGTCTGCACTCTGC-3’		
**DCH C**	F: 5’-CTCAGGTCTTTGCCCACTCA-3’	1155	55℃
	R: 5’-AGCTGACTCCTCCCAACAGT-3’		
**DCH D**	F: 5’-AACTAAGCATGAACTCCGCC-3’	1173	55℃
	R: 5’-TGGGCCAACAGGTGCAATTT-3’		

A total of 50 reference sequences from the family *Hepadnaviridae* were included together with the DCH Australia (AUS/2016/Sydney), Italy (ITA/2018/165 − 83) and Malaysian (DCH UPM) strains for the phylogenetic analysis. Evolutionary analyses were conducted using Molecular Evolutionary Genetics Analysis 7 (MEGA7) [30] software. Multiple sequence alignments were done for the best sequence match alignment. The evolutionary history was inferred using the Maximum Likelihood method. The percentage of trees in which the associated taxa clustered together is shown next to the branches. The tree reliability was evaluated using 1000 bootstrap replications. The tree was drawn to scale, with branch lengths measured in the number of substitutions per site. All positions containing gaps and missing data were eliminated.

### Serum biochemistry, FIV and FeLV tests

All sera from the shelter and pet cats were measured for serum biochemistry (ALT, ALP, urea, creatinine) using a VetTest Chemistry Analyzer (IDEXX, Washington, USA). The ALT and ALP results were classified as either normal (within normal limits) or abnormal (> 105% normal limits); the abnormality thresholds were 95 U/L for ALT and 84 U/L for ALP (cf. normal limits: ALT 10–90 U/L; ALP < 80 U/L). The serum samples were tested using the FeLV Ag/FIV Ab rapid test kit (GenBody, Korea) according to the manufacturer’s protocol.

### Data analysis

Statistical analyses were done using the Statistical Package for the Social Sciences Version 22, (SPSS Inc., USA). Prevalence data are reported as frequencies and percentages. Inferential statistical tests were carried out using χ-squared analysis and by evaluating the odds ratio (OR); 95% confidence intervals (CI) were calculated, where applicable, at a significance level of α = 0.05. The risk factors were analysed when the *p*-value for the likelihood ratio was ≤ 0.05. The χ-squared test was not applicable when one of the expected cell values was < 5. In these cases, Fisher’s exact test was used, with α = 0.05, to determine the association of the risk factors with the DCH-positive detection.

## Data Availability

Data will be available upon request from the corresponding author.

## Abbreviations

DCH: Domestic cat hepadnavirus
FIV: Feline immunodeficiency virus
FeLV: Feline leukemia virus
HBV: Human hepatitis B virus
ALT: Alanine transaminase
ALP: Alkaline phosphatase
UPM: Universiti Putra Malaysia
UVH: University Veterinary Hospital
FFPE: Formalin-fixed, paraffin-embedded
HCC: Hepatocellular carcinoma
